# *Artemia* Nauplii Enriched with Soybean Lecithin Enhances Growth Performance, Intestine Morphology, and Desiccation Stress Resistance in Yellow Drum (*Nibea albiflora*) Larvae

**DOI:** 10.3390/metabo15010063

**Published:** 2025-01-17

**Authors:** Zhenya Zhou, Pian Zhang, Peng Tan, Ruiyi Chen, Weihua Hu, Ligai Wang, Yuming Zhang, Dongdong Xu

**Affiliations:** 1 Fisheries College, Zhejiang Ocean University, Zhoushan 316022, China; 0403zzy@gmail.com (Z.Z.); zpupzp@163.com (P.Z.); 2 Key Laboratory of Mariculture and Enhancement, Zhejiang Marine Fisheries Research Institute, Zhoushan 316021, China; xx_cry@163.com (R.C.); huweihua9076@163.com (W.H.); wligaikuaile@126.com (L.W.); 3 Agro-Tech Extension Center of Guangdong Province, Guangzhou 510145, China; 13531694158@163.com

**Keywords:** soybean lecithin, *Artemia* nauplii, larvae, growth performance, intestine morphology

## Abstract

The inherent deficiency of phospholipids in *Artemia* limits its nutritional value as live prey for marine fish larvae. In our previous study, we optimized a phospholipid enrichment method by incubating *Artemia* nauplii with 10 g of soybean lecithin per m^3^ of seawater for 12 h, significantly enhancing their phospholipid content. **Purpose**: The present study evaluated the impact of this enrichment on yellow drum (*Nibea albiflora*) larvae, focusing on growth performance, intestinal morphology, body composition, weaning success, and desiccation stress resistance. **Methods**: The larvae (12 days post-hatching, dph) were fed either soybean lecithin-enriched (SL group) or newly hatched (NH group) *Artemia* nauplii for 10 days. **Results**: By the end of the experiment, the SL group exhibited a markedly greater body weight and standard length compared to the NH group (*p* < 0.05). This growth improvement was due to enhanced intestinal morphology, characterized by a significantly higher mucosal fold height, microvillus density, and microvillus length (*p* < 0.05). Intestinal RNA sequencing identified 160 upregulated and 447 downregulated differentially expressed genes (DEGs) in the SL group compared to the NH group. Soybean lecithin enrichment reduced the expression of lipogenesis-related genes (*fasn*, *scd*, *elovl1*) while upregulating lipid catabolism genes (*ppara*, *cpt1*, *cpt2*), indicating increased lipid breakdown and energy production. After a 5-day weaning period onto a commercial microdiet, the SL group continued to show significantly superior growth performance. In an afterward desiccation stress test, larvae from the SL group demonstrated significantly higher survival rates, potentially due to the decreased expression of intestinal cytokine genes (*ccl13*, *mhc1*, *mhc2*) observed in the RNA-seq analysis. **Conclusions**: This study highlights that feeding soybean lecithin-enriched *Artemia* nauplii enhances growth performance and desiccation stress in yellow drum larvae by promoting lipid catabolism, improving intestinal structure, and regulating immune responses.

## 1. Introduction

The successful rearing of marine fish larvae remains a critical challenge and a significant bottleneck in aquaculture. Due to their underdeveloped digestive systems, most marine fish larvae rely on live prey during early developmental stages, as they struggle to efficiently utilize formulated diets [1]. Among live prey, *Artemia* is widely used; however, its nutritional composition is often inadequate, lacking essential lipids like docosahexaenoic acid (DHA), eicosapentaenoic acid (EPA), and phospholipids—key nutrients for optimal larval growth and development [2,3]. Marine fish larvae have little or no ability to synthesize phospholipids de novo, making dietary supplementation critical during early development [4]. *Artemia* nauplii, capable of filtering and ingesting particles of suitable size, can be enriched with specific nutrients by adding enrichment materials to their culture medium [5]. This makes *Artemia* an effective vector for delivering essential nutrients tailored to the needs of marine larvae.

Phospholipid supplementation in the aquatic larvae stage offers several benefits, including the emulsification of dietary lipids [6], promotion of lipid absorption [7] and transport [8], and enhancement of digestive enzyme activity [9]. While the advantages of phospholipid-enriched microdiets for larval fish are well documented, only a little research has focused on enriching *Artemia* nauplii with phospholipids. Studies have demonstrated that *Artemia* metanauplii can be enriched with phospholipids through short-term incubations of approximately 4 h [10]. In contrast, *Artemia franciscana* nauplii was successfully enriched with soybean lecithin during an 18 h incubation at a concentration of 0.6 g/L seawater [11]. Although enrichment improves the nutritional profile of *Artemia*, it may have unintended effects, such as a reduction in nutritional quality under certain conditions [12]. Despite the known benefits of phospholipid enrichment, standardized and optimized protocols for effective soybean lecithin enrichment are still lacking, indicating the need for further research. Our previous study demonstrated that 10 g of soybean lecithin per m^3^ seawater, combined with a 12 h incubation, could effectively enrich *Artemia* nauplii, increasing their phospholipid content by 81% [13]. However, it remains unclear whether this enrichment strategy effectively supports marine larvae rearing. Traditional assessments of larval nutrition typically focus on growth metrics such as size, length, biomass, as well as morphological traits, and enzymatic activity. More recently, molecular biology techniques, including RNA sequencing with unique molecular identifier (UMI) technology, have complemented these approaches, providing deeper insights into the genetic mechanisms underlying development, metabolism, and immunity. These advanced tools allow for the precise classification of mRNA duplicates and offer robust insights into molecular responses to early feeding strategies [14].

The yellow drum (*Nibea albiflora*) is an important species for aquaculture and fishery enhancement on the coast of China, yet its larval-rearing practices remain underdeveloped. Current methods often fail to optimize larval growth and survival, highlighting the need for improved strategies. As the central organ responsible for nutrient digestion and absorption, the intestine plays a pivotal role in larval development, with effective digestion and nutrient uptake heavily reliant on a well-developed intestinal structure. Recent studies have demonstrated that dietary soybean lecithin significantly enhances intestinal development in yellow drum larvae, emphasizing the importance of refining nutritional approaches [15]. This study examines the effects of soybean lecithin enrichment on growth performance, intestinal morphology, weaning success, and desiccation stress resistance in yellow drum larvae fed either soybean lecithin-enriched or newly hatched *Artemia* nauplii. By integrating biochemical and molecular analyses, this study aims to advance nutritional strategies for promoting optimal larval development in yellow drum.

## 2. Materials and Methods

**Figure 1:**
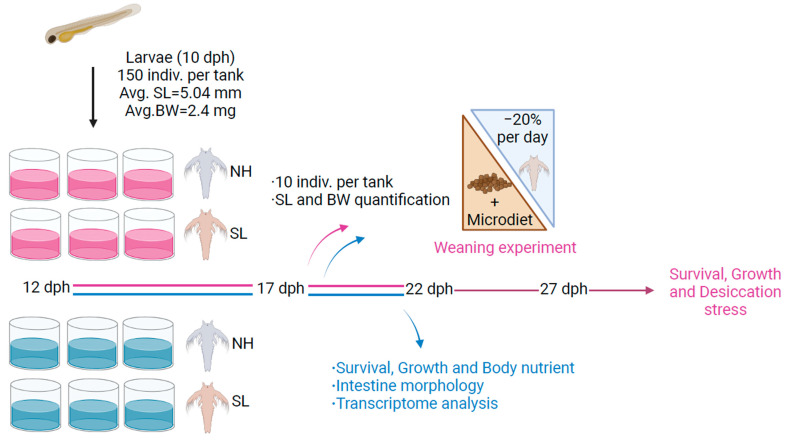
General workflow of the present study. The 12 post-hatching (dph) larvae were fed either soybean lecithin-enriched *Artemia* nauplii (SL) or newly hatched *Artemia* nauplii (NH). At the end of feeding stage 1 (17 dph), growth performance was analyzed using 10 larvae per tank (*n* = 6). By the end of feeding stage 2 (22 dph), larvae from three tanks per group were sampled to assess growth performance, body nutrient composition, and intestinal tissue morphology. Transcriptome analysis was performed to uncover the molecular mechanisms driving the observed outcomes. A weaning experiment was subsequently performed over five days, during which the proportion of *Artemia* nauplii was reduced by 20% daily, while microdiet quantities were adjusted based on larval consumption. Finally, a desiccation stress resistance test was conducted to evaluate larval stress tolerance. Avg. SL refers to the average standard length (SL) of larvae, while Avg. BW represents the average body weight (BW) of larvae.

In accordance with the Guide for the Care and Use of Laboratory Animals issued by the Ministry of Science and Technology of China, all experimental larvae in this study were handled with care. The experimental protocol was approved by the Committee on Ethics of Animal Experiments at Zhejiang Ocean University and adhered to the ethical principles and standards outlined by Metabolites. Before sampling, larvae were anesthetized with tricaine methanesulfonate (MS-222, Sigma, St. Louis, MO, USA) at a concentration of 10 mg/L. The general workflow of the present study is illustrated in Figure 1.

### 2.1. Nutritional Composition Analysis

The crude protein, crude lipid, moisture, and ash contents of raw ingredients and larval samples were analyzed in duplicate following the methods recommended by the Association of Official Analytical Chemists (AOAC) [16]. Moisture content was determined by drying samples until a stable weight was achieved. The crude protein content was determined using the bicinchoninic acid (BCA) assay method. Lipid extraction and quantification were performed using a Soxtec System HT (Soxtec 2055, FOSS Tecator, Sweden). Phospholipid content in larvae was assessed using the molybdenum blue colorimetric method, as described in a previous study [17]. Total lipids were extracted and purified using the method described in a previous study, and fatty acid methyl esters (FAMEs) were prepared following established protocols, with the fatty acid composition expressed as the molar percentage (mol %) of total identified fatty acids [18]. Amino acid content was analyzed according to the methodology detailed in our previous publication, with the results reported as a percentage of dry weight [13].

### 2.2. Soybean Lecithin Enrichment in Artemia Nauplii

As previously described [13], 10 g of soybean lecithin (Cargill, Germany) was accurately weighed and mixed with approximately 100 mL of fresh water at 50 °C. The mixture was thoroughly blended to achieve a uniform suspension, which was then diluted to a final volume of 500 mL by adding cold water. The resulting stock solution, containing 2% soybean lecithin (*w*/*v*), was stored at −20 °C. Newly hatched *Artemia* nauplii (~2 × 10^5^ individuals per liter) were transferred into the enrichment tank and enriched with soybean lecithin at approximately 28 °C for 12 h, using a dosage of 10 g/m^3^. After enrichment, the *Artemia* nauplii were collected and stored at 4 °C before feeding.

### 2.3. Larval Rearing

Yellow drum (*Nibea albiflora*) larvae were hatched at the Xixuan Fishery Science and Technology Island in Zhoushan, Zhejiang Province, China. After hatching, the larvae were cultured in a 40 m^3^ cement pool. From 3 to 10 days post-hatching (dph), they were initially fed rotifers (*Brachionus rotundiformis*), followed by co-feeding with rotifers and *Artemia* nauplii from 10 to 12 dph. At 10 dph, 1800 larvae were randomly distributed into 12 tanks, with 150 individuals per tank. To maintain consistent larval quality, deceased larvae were removed and replaced as necessary until 12 dph. At the start of 12 dph, a feeding trial was initiated with larvae averaging a standard length of 5.10 mm and a body weight of 2.04 mg. The tanks were divided into two groups, each with six replicates. The control group was fed newly hatched (NH) *Artemia* nauplii, and the experimental group (SL) was fed *Artemia* nauplii enriched with soybean lecithin. During the 10-day feeding trial, water conditions were maintained at a temperature of 28 ± 2 °C, pH 7.2–8.3, and a light/dark cycle of 16 h/8 h. The larvae were fed twice daily, at 7:00 and 18:00. To ensure precise feeding, *Artemia* nauplii were measured using a 3 mL Pasteur pipette, and equal amounts of thoroughly washed nauplii were added to maintain a concentration of 5 nauplii/mL in each tank. Tanks were cleaned twice daily by siphoning out waste, uneaten feed, and dead larvae. Mortality was carefully monitored and recorded during each cleaning session, with removed larvae identified and documented.

### 2.4. Sample Collection

Prior to the feeding trial, the initial body weight and standard length of the larvae were determined by averaging the weight and length of 50 randomly selected individuals. At the end of feeding stage 1 (17 dph), 20 larvae from each tank were randomly sampled to measure body weight and standard length. The larvae were anesthetized, carefully drained of seawater, and measured for total weight and length. At the end of feeding stage 2 (22 dph), three tanks were randomly selected from each treatment group. All larvae were anesthetized and sacrificed to record their body weight and standard length. A total of 10 larvae per tank were sacrificed for mid-intestinal tissue collection and RNA extraction. Intestinal samples were preserved in RNAlater (Thermo Fisher Scientific, Waltham, MA, USA) and stored at −20 °C for subsequent processing. Mid-intestinal tissues from 10 larvae per tank were preserved in a 4% paraformaldehyde (PFA) solution for hematoxylin and eosin (H&E) section analysis. A total of 60 histological sections were randomly selected, with 30 sections analyzed per treatment group to ensure representative sampling. Additionally, 10 larvae per tank were randomly selected for electron microscopy analysis, with thin sections of the mid-intestine prepared for this purpose. The remaining larvae were collected, frozen at −20 °C, and later analyzed for proximate nutritional composition, fatty acid profiles, and amino acid content.

### 2.5. Weaning Experiment and Desiccation Stress Challenge

The weaning experiment lasted 5 days. Larvae were fed a mixture of *Artemia* nauplii and a commercial microdiet (Hayashikane Co., Ltd., Shimonoseki, Yamaguchi, Japan). The proportion of Artemia nauplii was reduced by 20% daily relative to the initial amount, meaning that on the first day of feed transition, the *Artemia* nauplii accounted for 80% of the original amount, 60% on the second day, and so on. The amount of microdiet was increased according to larval consumption. The weaning process was completed at the start of day 5 (27 dph). Larval mortality was recorded twice daily throughout the weaning experiment.

Desiccation stress refers to the exposure of fish to air for a specific duration, simulating conditions that induce dehydration and oxidative stress. This stress test is commonly used to evaluate the resilience of fish to environmental challenges, particularly in aquaculture settings. The median lethal desiccation time (LD50) refers to the duration of desiccation at which 50% of the test larvae succumbed under controlled experimental conditions. Preliminary experiments were performed to establish the desiccation stress challenge duration required to determine the LD50. At 27 dph, all larvae per tank were placed in a mesh net and exposed to air for exactly 210 s. The larvae were then immediately returned to their respective tanks. Mortality was monitored over the following 12 h, and deceased larvae were collected and counted. All the larval standard length and body weight were obtained after the desiccation stress challenge.

### 2.6. Intestinal Structure and Ultrastructure Analysis

#### 2.6.1. Hematoxylin and Eosin (H&E) Section Analysis

Mid-intestinal samples were sectioned into 6 μm slices and stained with H&E following standard protocols [19,20]. Imaging was performed using a Panoramic 250 FLASH digital pathology system (3DHISTECH, Budapest, Hungary), and parameters such as the mucosal fold height, muscular thickness, and perimeter ratio were measured with CaseViewer software (v2.4.0.119028) [21]. A total of 60 tissue sections were analyzed, with 30 sections examined per treatment group.

#### 2.6.2. Ultrastructure Analysis

Ultrathin sections were stained with 2% uranyl acetate and 0.2% lead citrate, and then examined using a transmission electron microscope (TEM) (JEOL, JEM-1200, Tokyo, Japan) operating at 80 kV. For scanning electron microscopy (SEM), samples were fixed in 1% osmium tetroxide (OsO_4_), dehydrated through a graded ethanol series (30% to 100%), critical-point dried, and sputter-coated with gold. The SEM imaging was conducted using an SU-8100 microscope (HITACHI, Tokyo, Japan). A total of 40 ultrathin section images were captured and analyzed using Image-Pro Plus 6.0 software (Media Cybernetics, Rockville, MD, USA). Microvilli density and length were quantified according to the methodology described by Gu et al. [22].

### 2.7. Unique Molecular Identifier (UMI) RNA-Seq and Bioinformatics Analysis

Total RNA from larval mid-intestine tissues (NH and SL groups) was extracted using TRIzol reagent (Invitrogen, Carlsbad, CA, USA). RNA quality, purity, and integrity were assessed via agarose gel electrophoresis, NanoPhotometer^®^, Qubit^®^ Fluorometer, and Agilent Bioanalyzer 2100. Library construction and sequencing were carried out with assistance from Novogene Co., Ltd. (Beijing, China). A modified protocol, based on our previous study, was employed for RNA-seq library preparation, quality control, and downstream analyses [23]. During second-strand cDNA synthesis, UMI tags and sequencing adapters were added. Sequencing was performed on the Illumina HiSeq 2500 platform (Illumina, San Diego, CA, USA). Differential expression analysis identified significant DEGs based on a q-value < 0.05 and a fragments-per-kilobase-per-million (FPKM) fold-change greater than 2.

### 2.8. DEGs Validation

Total RNA extracted for Illumina sequencing was utilized to synthesize complementary DNA (cDNA). Reverse transcription was carried out using the PrimeScript RT Reagent Kit with a gDNA Eraser (Takara Bio Inc., Kusatsu, Shiga, Japan), according to the manufacturer’s instructions. *18S rRNA* and *α-tubulin* served as internal controls for normalizing gene expression levels. The expression profiles of 16 target genes were validated through real-time quantitative PCR (qRT-PCR), with primer sequences listed in Table S1. qRT-PCR was conducted on an ABI Step One Plus Real-Time PCR System (Applied Biosystems, Foster City, CA, USA), using the program settings described in our previous study [23]. The comparative CT method (ΔΔCT) was applied to calculate relative gene expression levels [24].

### 2.9. Calculations and Data Statistics

The following variables were calculated:

Final body weight (FBW, g) = W_t_/N_t_

Survival rate (SR, %) = 100 × N_t_/N_0_

Survival rate after desiccation stress (%) = 100 × larvae survival quantity/number of larvae for the stress test

where W_t_ represents the total final body weights of the larvae; N_t_ denotes the final larval population, N_0_ is the initial larval population in each tank, and t refers to the duration of the experiment in days.

All data were analyzed using GraphPad (Version 10.3.0, Windows, San Diego, CA, USA) and are expressed as the mean ± standard deviation (S.D.). The data between the two groups were analyzed and compared by independent *t*-tests. Differences were considered significant at a level of *p* < 0.05. The Levene test was employed to assess the normality and homogeneity of variances. DEG association networks were predicted using String (https://string-db.org, accessed on 3 September 2024) and visualized with Cytoscape (http://www.cytoscape.org, accessed on 3 September 2024).

## 3. Results

### 3.1. Larval Survival and Growth Performance

**Figure 2:**
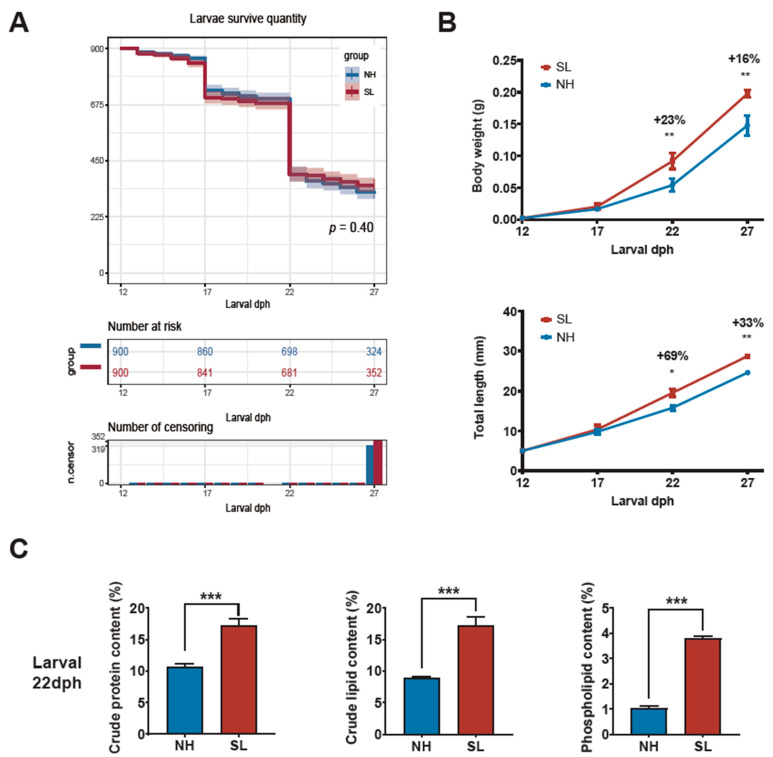
Survival, growth performance, nutrient composition in yellow drum larvae. (**A**) Larval survival rate at the end of feeding stage 1 (17 dph), feeding stage 2 (22 dph), and after weaning experiment (27 dph). (**B**) Larval body weight and standard length. (**C**) Crude lipid crude protein and phospholipid content (%, dry weight) of larvae at the end of feeding stage 2 (22 dph) and after weaning experiment (27 dph). * *p* < 0.05, ** *p* < 0.01, *** *p* < 0.001.

No significant difference in survival rates was observed between the groups at the end of the feeding trial (22 dph) or after the weaning experiment (27 dph) (*p* = 0.40, Figure 2A). At the end of feeding stage 1 (17 dph), larval body weight and standard length were similar between the NH and SL groups (*p* > 0.05, Figure 2B). However, by the end of stage 2 (22 dph), the SL group exhibited a significantly higher body weight (*p* < 0.01) and standard length compared to the NH group (*p* < 0.05) (Figure 2B). Similarly, after the weaning experiment (27 dph), the SL group continued to show a significantly higher body weight and standard length (*p* < 0.01, Figure 2B).

### 3.2. Larval Proximate Nutrients, Fatty Acids, and Amino Acids Analysis

At the end of feeding stage 2 (22 dph), analysis of larval nutrient composition revealed significantly higher crude protein and crude lipid contents in the SL group compared to the NH group (*p* < 0.001, Figure 2C). However, these differences were no longer significant after the weaning experiment (27 dph) (*p* > 0.05, Figure 2C). Notably, larvae fed soybean lecithin-enriched *Artemia* nauplii exhibited significantly higher phospholipid content at both the end of feeding stage 2 and after the weaning experiment (*p* < 0.001, Figure 2C).

Larvae in the SL group had a significantly higher proportion of monounsaturated fatty acids (MUFAs) compared to those in the NH group (*p* < 0.05). No significant differences were observed in the proportions of saturated fatty acids (SFAs), n-3 polyunsaturated fatty acids (n-3 PUFAs), or n-6 polyunsaturated fatty acids (n-6 PUFAs) (*p* > 0.05, Table 1). There were no significant differences in the levels of essential amino acids (EAAs), nonessential amino acids (NEAAs), or total amino acids (TAAs) between the groups (*p* > 0.05, Table 2).

### 3.3. Larval Survival After Desiccation Stress Challenge

**Figure 3:**
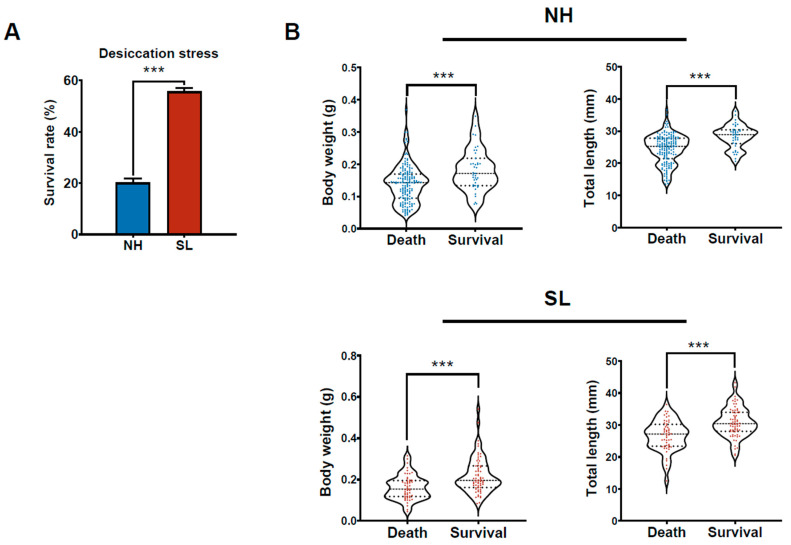
Desiccation stress challenge. (**A**) Survival rate of larvae after the desiccation stress challenge. (**B**) Comparison of body weight and standard length between surviving and dead larvae. *** *p* < 0.001.

The survival rate of the SL group after the desiccation stress challenge was significantly higher than that of the NH group (*p* < 0.001, Figure 3A). Additionally, statistical analysis revealed that the body weight and standard length of surviving larvae in both groups were significantly greater than those of the deceased larvae (*p* < 0.001, Figure 3B). To assess the influence of growth performance and SL treatment on survival under desiccation stress, a logistic regression analysis was performed, incorporating body weight and SL treatment as predictors. The results showed that the survival rate after desiccation stress was significantly affected by body weight (*p* < 0.001), whereas the direct effect of SL treatment, independent of body weight, was not statistically significant (*p* = 0.537).

### 3.4. Soybean Lecithin Enrichment on Larval Intestinal Morphology

**Figure 4:**
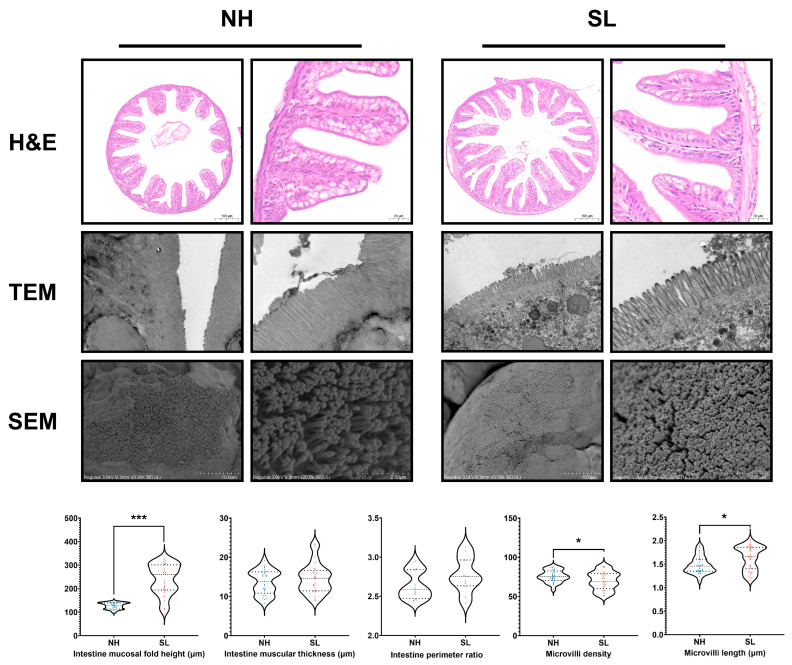
Intestinal tissue morphology and ultrastructure analysis. Violin plots illustrate the perimeter ratio, muscular thickness, and mucosal fold height of intestinal tissues. Scanning electron microscopy (SEM) images reveal the surface ultrastructure of intestinal tissues, while transmission electron microscopy (TEM) images display microvillus morphology, including quantified microvillus density and length. * *p* < 0.05, *** *p* < 0.001; otherwise, differences were not significant (*p* > 0.05).

Feeding larvae with soybean lecithin-enriched *Artemia* nauplii significantly enhanced intestinal morphology compared to the NH group, as indicated by a significant increase in the mid-intestine fold height (*n* = 25, *p* < 0.001, Figure 4). Electron microscopy analysis supported the morphology improvements, showing significantly greater intestinal microvillus length (*n* = 35, *p* < 0.05) and density (*n* = 25, *p* < 0.05) in the SL group (Figure 4).

### 3.5. Intestine RNA-Seq Quality Control, Mapping, and Bioinformatics Analysis

**Figure 5:**
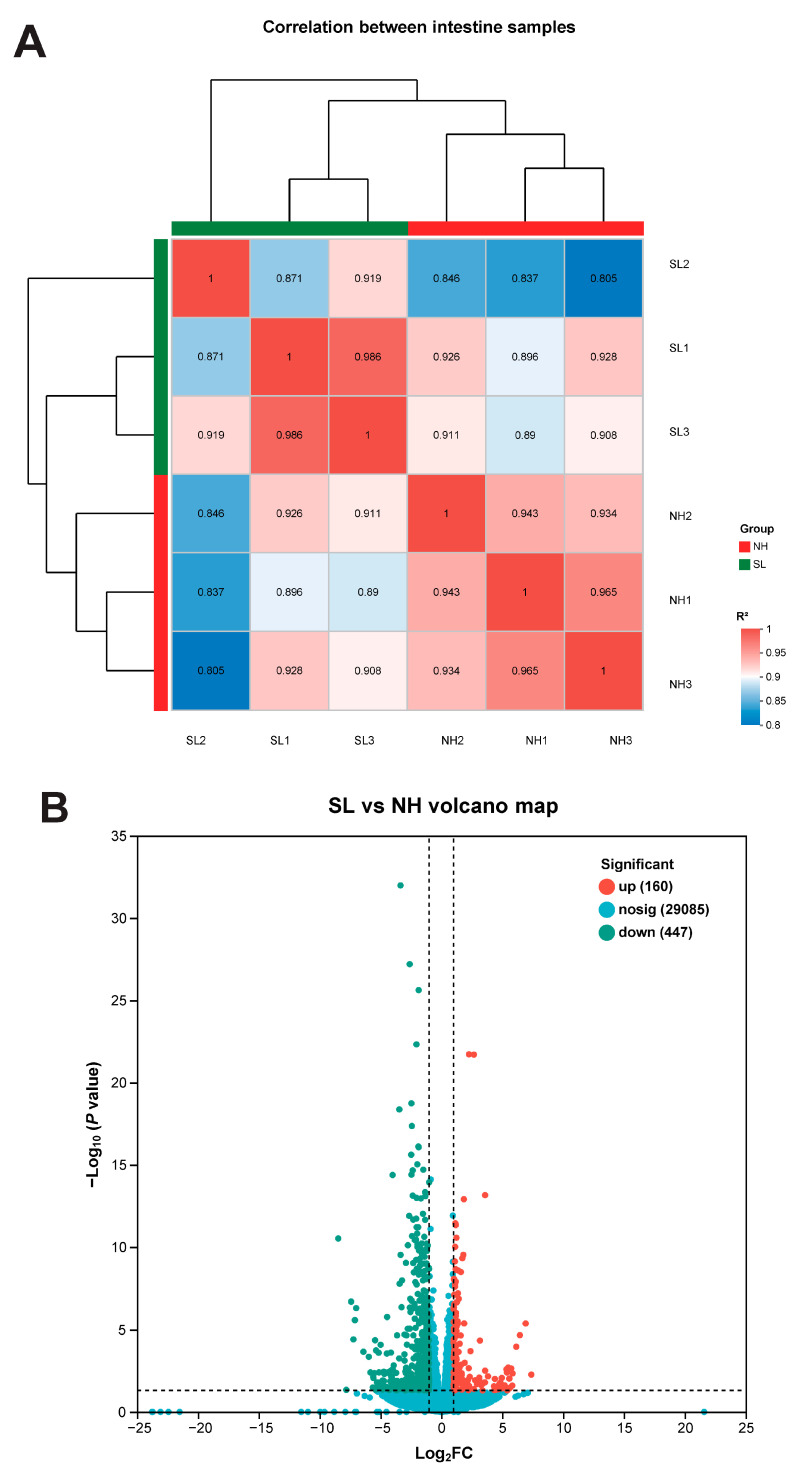

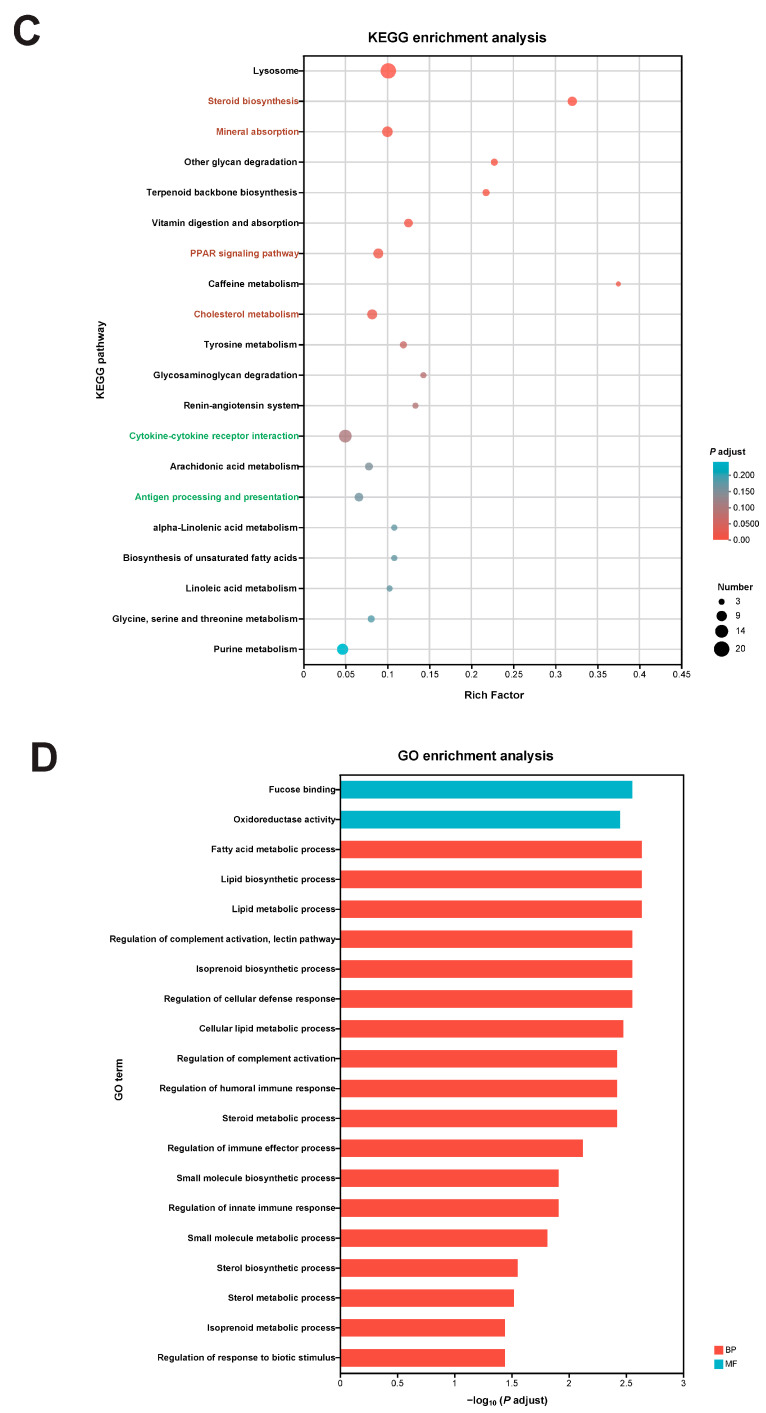
Transcriptome analysis of larval intestine tissues. (**A**) Pearson’s correlation matrix of intestinal tissue samples. (**B**) Volcano plot depicting DEGs between groups. (**C**) Enrichment analysis of DEGs for Kyoto Encyclopedia of Genes and Genomes (KEGGs) pathways. (**D**) Enrichment analysis of DEGs for Gene Ontology (GO) terms.

The correlation analysis of intestinal tissue samples from the SL and NH groups is shown in Figure 5A. A total of 29,692 genes were identified in the intestinal tissue samples, with 160 upregulated differentially expressed genes (DEGs) and 447 downregulated DEGs (Figure 5B). The top 20 Kyoto Encyclopedia of Genes and Genomes (KEGGs) pathways in the intestinal tissue are shown in Figure 5C. The most enriched KEGG pathways were lysosome, steroid biosynthesis, and mineral absorption, with pathways such as glycan degradation, terpenoid backbone biosynthesis, vitamin digestion and absorption, PPAR signaling, and cholesterol metabolism also involved.

**Figure 6:**
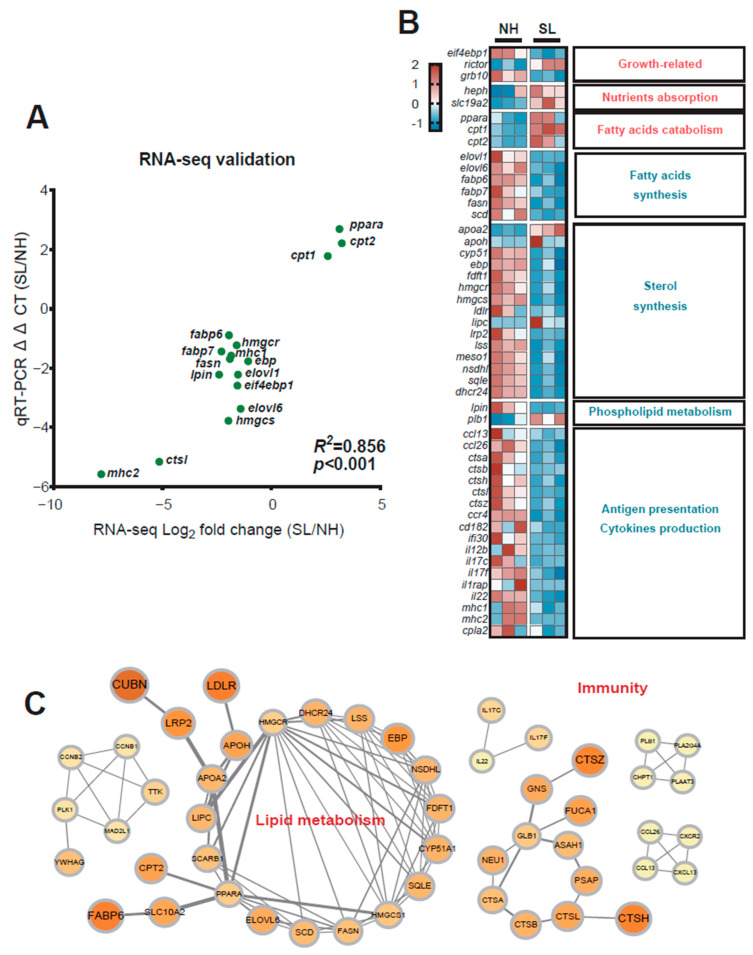
DEGs encoding protein–protein interaction (PPI) networks. (**A**) Validation of RNA-seq results using qRT-PCR for selected DEGs. (**B**) Heatmap of selected DEGs between treatments. (**C**) Network of DEGs related to lipid metabolism and immunity pathways in intestinal tissue.

To validate the reliability of the DEGs identified by RNA sequencing, qRT-PCR analysis was performed on selected genes. The results showed consistent expression trends between the RNA-seq and qRT-PCR data (Log_2_-fold change vs. ΔΔCt). Pearson correlation analysis revealed an *R*^2^ value of 0.856, with a *p* value less than 0.001 (Figure 6A). These findings confirm the reliability of the UMI-RNA data used in this study. DEGs related to growth (*eukaryotic translation initiation factor 4E-binding protein 1* [*eif4ebp1*], *rapamycin-insensitive companion of mTOR* [*rictor*], *growth factor receptor-bound protein 10* [*grb10*]), lipid catabolism (*peroxisome proliferator-activated receptor α* [*ppara*], *carnitine palmitoyltransferase 1* [*cpt1*], *carnitine palmitoyltransferase 2* [*cpt2*]), fatty acid synthesis (*fatty acid synthase* [*fasn*], *stearoyl-CoA desaturase* [*scd*], *very long chain fatty acid elongase 1* [*elovl1*], *fatty acid-binding protein 6* [*fabp6*]), sterol synthesis (*3-hydroxy-3-methylglutaryl-coenzyme a reductase* [*hmgcr*], *hydroxymethylglutaryl-CoA synthase* [*hmgcs*], *squalene monooxygenase* [*sqle*], *Δ(24)-sterol reductase* [*dhcr24*]), and immunity (*C-C motif chemokine 13* [*ccl13*], *C-C motif chemokine 26* [*ccl26*], *procathepsin l* [*ctsl*], *major histocompatibility complex class I-related gene protein*; [*mhc1*], *major histocompatibility complex class II-related gene protein* [*mhc2*]) were identified in the intestinal tissue (Figure 6B). The predicted protein–protein interaction (PPI) networks for DEGs in the intestinal tissue are shown in Figure 6C. The interactions of genes related to lipid metabolism, such as *ppara*, *cpt1*, *cpt2*, *hmgcr*, and *hmgcs*, are displayed, along with interactions of genes related to immunity, including *interleukin-22* (*il22*), *interleukin-17f* (*il17f*), *interleukin-17fc* (*il17c*), *procathepsin a* (*ctsa*), *procathepsin b* (*ctsb*), and *procathepsin h* (*ctsh*).

## 4. Discussion

Our previous study highlighted the critical role of phospholipids in enhancing growth performance during the larval stage of yellow drum fed with microdiets. These benefits are primarily attributed to improvements in intestinal morphology and phospholipid catabolism, which enhance nutrient absorption and spare amino acids for protein synthesis, ultimately supporting overall larval growth [17]. Based on these findings, the present study demonstrated that enriching *Artemia* nauplii with soybean lecithin prior to feeding yellow drum larvae significantly improved growth performance, with a 23% increase in body weight and a 59% increase in standard length after a 10-day feeding trial. This is consistent with recent research that early dietary supplementation with soybean lecithin promotes the growth of yellowfin seabream (*Acanthopagrus latus*) larvae [25]. However, while the present study utilized 10 g of soybean lecithin per ton of seawater to enrich *Artemia* nauplii for 12 h, Morshedi et al. employed a comprehensive lipid emulsion containing 48–72 g of soybean lecithin. The variation in results could result from differences in environmental factors such as soybean lecithin enrichment duration, the composition of nutritional fortifiers, and *Artemia* enrichment density. Despite these discrepancy results, studies on Neotropical green terror cichlid (*Aequidens rivulatus*) larvae [11] and common octopus (*Octopus vulgaris*) paralarvae [10] support the efficacy of enriching live prey with soybean lecithin to improve larval rearing performance by enhancing *Artemia*’s nutritional value.

Optimal growth is closely linked to the accumulation of essential body nutrients. In this study, larvae in the SL group exhibited significantly higher levels of crude protein, crude lipid, and phospholipids compared to the control group, likely due to the enhanced nutrient absorption facilitated by improved mucosal fold height, microvilli density, and length [9]. These results align with previous studies showing that dietary phospholipid supplementation improves larval intestinal morphology and nutrient absorption efficiency [23,26,27]. However, the larger size of SL group larvae may have contributed to these structural improvements, as larger fish tend to exhibit more developed intestines. While histological samples were randomly selected, the absence of size normalization limits the ability to disentangle size effects from the direct influence of SL enrichment. Despite this limitation, SL supplementation appears to directly support mid-intestinal development by optimizing the intestinal environment for nutrient uptake.

The beneficial effects of soybean lecithin on intestinal morphology are largely attributed to its involvement in energy metabolism. Studies have shown that energy depletion is closely linked to intestinal damage and structural deterioration [28,29]. In the SL group, the expression of key genes involved in mitochondrial fatty acid β-oxidation, including *ppara*, *cpt1*, and *cpt2*, was significantly upregulated. *Ppara*, a central regulator of lipid metabolism in vertebrates, activates *cpt1*, which facilitates the transfer of acyl groups from long-chain fatty acid-CoA conjugates to carnitine—a critical step in mitochondrial fatty acid oxidation [30,31]. Previous research in zebrafish (*Danio rerio*) has shown that *cpt1* deficiency reduces mitochondrial fatty acid β-oxidation [32]. The upregulation of these genes in the SL group suggests enhanced energy production, meeting the high energy demands of rapidly proliferating and renewing intestinal cells [33]. These findings highlight soybean lecithin as an essential energy source for enterocyte proliferation and repair, thereby improving intestinal structure, nutrient digestion, and absorption.

A well-developed intestinal morphology is essential not only for efficient nutrient digestion and absorption but also for protecting fish from external threats [34]. The intestine acts as both a physical and immunological barrier, preventing harmful substances and pathogens from entering systemic circulation. In this study, RNA-seq analysis revealed that larvae fed soybean lecithin-enriched *Artemia* nauplii exhibited significantly lower mRNA expression of chemokines such as *ccl13* and *ccl26*, as well as other immune-related genes, including *mhc1* and *mhc2*, in the mid-intestine. CCL13 and CCL26 are cytokines critical for immune regulation, particularly in immune cell migration under both inflammatory and homeostatic conditions. They facilitate leukocyte recruitment to sites of infection or injury, as well as immune cell activation and differentiation in fish species [35,36]. The reduced chemokine expression observed in the SL group suggests that soybean lecithin helps promote a balanced immune response by minimizing unnecessary inflammatory signaling while maintaining an effective pathogen defense. MHC I and MHC II molecules play crucial roles in the immune system by presenting antigens to T cells, with MHC I primarily involved in presenting intracellular antigens and MHC II in extracellular antigen presentation. The observed low expression of *mhc1* and *mhc2* in this study may be indicative of a reduced inflammatory response, likely reflecting a more balanced immune state under the experimental conditions. This possibility highlights the need for additional experiments to elucidate the specific contributions of these molecules to immune modulation in the intestine. Furthermore, previous studies have shown that phospholipids, such as those found in soybean lecithin, enhance intestinal barrier function by stabilizing epithelial cell membranes and reinforcing tight junction integrity [37]. This improved barrier likely reduces antigen exposure to intestinal immune cells, which may contribute to the observed lower chemokine expression. Future studies incorporating functional assays, such as measurements of inflammatory cytokine levels or immune cell recruitment, are warranted to substantiate the broader implications of reduced chemokine expression on immune modulation.

Phospholipid supplementation enhances the ability of larvae to withstand environmental stress, as demonstrated in this study by the significantly higher survival rates of larvae fed soybean lecithin-enriched *Artemia* nauplii following a desiccation challenge. However, logistic regression analysis revealed that the improved survival rates were primarily attributed to enhanced growth performance rather than a direct, independent effect of SL treatment. Larger and more developed larvae demonstrated greater stress tolerance, highlighting the critical role of growth in physiological resilience. These findings align with Morshedi et al., who reported a positive correlation between growth performance and stress resistance in yellowfin seabream larvae exposed to air and osmotic stress [25]. The enhanced growth and stress tolerance observed in SL-fed larvae may also be linked to the modulation of inflammatory responses. Inflammatory processes, when excessive, can impair intestinal integrity and redirect energy from growth and development to immune defense [38]. In this study, soybean lecithin-enriched diets likely helped suppress unnecessary inflammatory signaling, as indicated by the reduced expression of inflammatory cytokine genes. This anti-inflammatory effect likely contributed to maintaining intestinal health by stabilizing epithelial cell membranes and preserving tight junction integrity, thereby reducing antigenic exposure and the inflammatory burden [39]. This dual benefit underscores the importance of phospholipids in promoting both growth and stress adaptation during early development.

## 5. Conclusions

Feeding trials and transcriptome analysis demonstrated the positive impact of soybean lecithin-enriched *Artemia* nauplii on growth performance, weaning success, intestinal morphology, and the underlying molecular mechanisms in yellow drum larvae. The key findings are summarized as follows:

(1) Soybean lecithin enrichment significantly enhanced yellow drum larval growth performance at the end of the 10-day trial and after the weaning experiment.
(2) Soybean lecithin-enriched *Artemia* nauplii enhanced intestinal morphology and promoted better nutrient absorption.
(3) Soybean lecithin supplementation increased survival rates under desiccation stress and regulated genes related to growth, lipid metabolism, and immunity, supporting overall larval health and development.

## Figures and Tables

**Table 1:** Fatty acid profile of 22-dph yellow drum larvae (%, identified fatty acids).

Fatty Acid	Larvae Body	
	NH	SL
C14:0	2.45 ± 0.49	2.22 ± 0.06
C15:0	0.35 ± 0.02	0.35 ± 0.01
C16:0	14.02 ± 0.65	13.46 ± 0.46
C17:0	0.51 ± 0.30	0.57 ± 0.03
C18:0	3.97 ± 1.50	4.65 ± 0.06
C24:0	N.A.	0.05 ± 0.09
∑SFAs ^1^	21.31 ± 1.89	21.31 ± 0.41
C14:1	0.44 ± 0.12	0.44 ± 0.01
C16:1	10.92 ± 1.53	10.81 ± 0.27
C17:1	0.22 ± 0.02	0.13 ± 0.01
C18:1n−9t ^2^	0.33 ± 0.05	0.34 ± 0.01
C18:1n−9c ^3^	32.52 ± 3.00	36.06 ± 1.05
C20:1n−9	N.A.	0.13 ± 0.01
∑MUFAs ^4^	44.35 ± 1.48 ^b^	47.91 ± 1.17 ^a^
C18:2n−6t	N.A.	0.08 ± 0.07
C18:2n−6c	8.32 ± 0.37	8.57 ± 0.19
C18:3n−6	0.51 ± 0.22	0.62 ± 0.11
C20:4n−6	2.50 ± 0.10	1.95 ± 0.17
∑n-6 PUFAs ^5^	11.32 ± 0.11	11.23 ± 0.31
C18:3n−3	8.31 ± 1.50	7.76 ± 0.51
C20:5n−3 (EPA)	13.26 ± 1.72	10.82 ± 0.82
C22:6n−3 (DHA)	0.54 ± 0.03 ^a^	0.32 ± 0.04 ^b^
C22:5n−3	0.47 ± 0.07	0.20 ± 0.17
∑n−3 PUFAs ^6^	22.58 ± 3.15	19.10 ± 1.25
∑n−3 LCPUFAs ^7^	14.27 ± 1.66	11.34 ± 0.77
C20:2	N.A.	0.03 ± 0.05
C22:2	0.43 ± 0.61	0.41 ± 0.11

Values are presented as mean ± S.D. (*n* = 3). Within a row, values with a superscript mean significantly different between experimental treatments (*p* < 0.05). ^1^ SFAs: saturated fatty acids. ^2^ c: cis-fatty acids. ^3^ t: trans-fatty acids. ^4^ MUFAs: monounsaturated fatty acids. ^5^ n−6 PUFAs: n−6 polyunsaturated fatty acids. ^6^ n−3 PUFAs: n-3 polyunsaturated fatty acids. ^7^ n−3 LC-PUFAs: n-3 long-chain polyunsaturated fatty acids.

**Table 2:** Amino acid content (g/100 g, dry weight) of 22 dph yellow drum larvae.

Amino Acids	Larvae Body	
	NH	SL
Arginine	3.27 ± 0.08	3.15 ± 0.09
Histidine	0.98 ± 0.03	0.97 ± 0.03
Isoleucine	2.00 ± 0.03 ^b^	1.87 ± 0.04 ^a^
Leucine	3.99 ± 0.09	3.83 ± 0.10
Lysine	4.20 ± 0.07 ^b^	4.96 ± 0.11 ^a^
Methionine	1.07 ± 0.29	1.11 ± 0.24
Phenylalanine	2.46 ± 0.04	2.41 ± 0.07
Threonine	2.53 ± 0.04 ^a^	2.40 ± 0.04 ^b^
Valine	2.54 ± 0.04 ^a^	2.42 ± 0.06 ^b^
EAAs ^1^	23.06 ± 0.58	22.12 ± 0.69
Alanine	3.47 ± 0.10	3.40 ± 0.06
Asparagine	5.43 ± 0.09	5.22 ± 0.10
Glutamic acid	8.30 ± 0.18	7.92 ± 0.20
Glycine	3.44 ± 0.10	3.26 ± 0.11
Proline	2.36 ± 0.04	2.28 ± 0.05
Serine	2.60 ± 0.03	2.47 ± 0.09
Tyrosine	1.84 ± 0.04	1.74 ± 0.05
NEAAs ^2^	27.44 ± 0.52	26.30 ± 0.63
TAAs ^3^	50.51 ± 1.09	48.42 ± 1.32

Values are presented as mean ± S.D. (*n* = 3). Within a row, values with a superscript mean significantly different between experimental treatments (*p* < 0.05). ^1^ EAAs: essential amino acids. ^2^ NEAAs: nonessential amino acids. ^3^ TAAs: total amino acids.

## Data Availability

Raw data were generated at the Zhejiang Marine Fisheries Research Institute. Derived data supporting the findings of this study are available from the corresponding author (Peng Tan) upon reasonable request.

## Supplementary Materials

The following supporting information can be downloaded at: https://www.mdpi.com/article/10.3390/metabo15010063/s1, Table S1: Primers used for qRT-PCR.
