# Supplementary material for: Artemia Nauplii Enriched with Soybean Lecithin Enhances Growth Performance, Intestine Morphology, and Desiccation Stress Resistance in Yellow Drum (Nibea albiflora) Larvae
Source: Metabolites. 2025 Jan 17;15(1):63. doi: 10.3390/metabo15010063 (PMC11767900; doi:10.3390/metabo15010063)
Supplement: Supplementary file 1 [file metabolites-15-00063-s001.zip › metabolites-3384903-supplementary.pdf]

Table S1. Primers used for qRT–PCR.

Primer Name	Primer Sequences (Forward Primer / Reverse Primer)	Product Size (bp)	PCR Efficiency (%)
cpt1-F/R	TTTCGCTTTCAACGAGGAGT / CTTTTCCACATGGGCTTGT	212	96
cpt2-F/R	CACCTGTTTGCCATGCGATA / GGGTAGCTGGACACGTTACA	224	100
ctsl-F/R	TGGCTCAACAATCGCAAACT / GTGACGTATCCCTTGTCCCT	245	101
ebp-F/R	TGTCATTGAGGGCTGGTTCT / CCAAAAGCTGAATGGTCCCC	175	98
eif4ebp1-F/R	AAGTTCCTCCTCCAGTGTCG / CGCACAGACACGTTTACCTG	189	96
elovl1-F/R	TGTTGGAGGAATGGGCTCTT / TACTGGCTGATGTGAACGGA	180	97
elovl6-F/R	ATGGGCTGTGTGGTCAACTA / CGTGGGTTTACTCGCTCTTC	202	95
fabp6-F/R	ATCCAACAAACGCCAAGGTC / GCAGATCTCAGAGGTGTGGT	155	99
fabp7-F/R	GTGGTGAAAACCCTGAGCAC / CAAATCTGGTCTCCTTGCCG	166	102
fasn-F/R	CTGGCTTGGTTGTGTCTTCC / GATTGGCCTCCTTGATGCAG	234	103
hmgcr-F/R	CGTCTGTTCCGTATGCATGG / ATTGAGGCCTGTGAGCTCTT	249	100
hmgcs-F/R	GCCTCACTCATTGCACAACA / CCTTCGTCCTGGAGTCAAGT	193	100
lpin-F/R	TTGTCCAGCCTGTGAACCAT / AGGACGTTGTTACAGGAGGG	244	99
mhc1-F/R	GAAGCGCTTGAACAAAACGG / CTGAGGTTTTGGTGCGATCC	169	98
mhc2-F/R	CTGGTTTCTATCCGGCTCCT / CACTGTGCAGCTGTAAACGT	167	96
ppara-F/R	GCAGTTGGACCAGGAAATGT/ CAGTGAGTCTGATGGCAGGA	217	101
18S rRNA -F/R	CTGCACGGACAGAAACTCAA / CTCTTTAGCCCCCTCTGCTT	167	96
α-tubulin-F/R	AGGTGGGCATCAACTACCAG / TGAGAACTCTCCCTCCTCCA	203	99

cpt1: *carnitine palmitoyltransferase 1;* ***cpt2***: *carnitine palmitoyltransferase 2;* ***ctsl***: *procathepsin l;* ***ebp***: *3-beta-hydroxysteroid-Δ8,Δ7-isomerase;* ***eif4ebp1***: *eukaryotic translation initiation factor 4E-binding protein 1;* ***elovl1***: *very long chain fatty acid elongase 1;* ***elovl6***: *very long chain fatty acid elongase 6;* ***fabp6***:

fatty acid-binding protein 6; ***fabp7****: fatty acid-binding protein 7;* ***fasn****: fatty acid synthase;* ***hmgcr****: 3-hydroxy-3-methylglutaryl-coenzyme a reductase;* ***hmgcs****: hydroxymethylglutaryl-CoA synthase;* ***lpin****: lipid phosphate phosphohydrolase;* ***mhc1****: major histocompatibility complex class I-related gene protein;* ***mhc2****: major histocompatibility complex class II-related gene protein;* ***ppara****: peroxisome proliferator-activated receptor α;* ***18S rRNA*** and ***α-tubulin*** are internal reference genes.
